# Exit strategies for health interventions in low- and middle-income countries: a systematic review

**DOI:** 10.1186/s44263-025-00182-6

**Published:** 2025-07-21

**Authors:** Maria Milenova, S’thembile Thusini, Petra C. Gronholm, Tatiana Taylor Salisbury

**Affiliations:** 1 Health Service and Population Research Department, Institute of Psychiatry, Centre for Global Mental Health, King’s College London, Psychology & Neuroscience, London, UK; 2 Health Service and Population Research Department, Institute of Psychiatry, King’s College London, Psychology & Neuroscience, London, UK; 3 Department of Population Health, London, School of Hygiene & Tropical Medicine , Centre for Global Mental Health, London, UK

**Keywords:** Exit strategies, Global health, ODA, International development, Sustainability, Systematic review, LMIC

## Abstract

**Background:**

Sustaining the benefits of externally funded interventions in low- and middle-income countries (LMICs) remains a concern in global health. As many initiatives depend on time-limited official development assistance (ODA) or philanthropic funding, exit strategies are increasingly recognised as key to ensuring positive impact. This systematic review examines how exit strategies have been conceptualised and implemented across a range of global health initiatives. We aim to identify, categorise and analyse the components of exit strategies during or after the implementation of health interventions in LMICs and their effectiveness. The results of this review will inform future global health programme design and research, including mental health.

**Methods:**

Database searches were conducted in Embase, Scopus, PubMed, PsycINFO (Elsevier), HMIC Health Management Information Consortium, MEDLINE, Social Policy and Practice, Web of Science and Global Health. The latest searches were run in January 2023. Data on study characteristics were descriptively synthesised. Extracted data regarding exit strategy processes and components were analysed using thematic synthesis.

**Results:**

Twenty-three articles (reflecting 22 studies) were identified for inclusion. Within these, eight key components of successful exit strategies were identified: (1) shared principles and values, (2) resource stability, (3) operational linkages, (4) local champions, (5) staff care and capacity, (6) leadership promotion, (7) mentoring and evaluation and (8) context-sensitive flexibility of exit. The studies showcased the complexity and interdependent nature of exit strategies in varied health contexts and provided insights into effective processes for sustained implementation.

**Conclusions:**

This review highlights the importance of planning for sustainability from the outset of health programmes in LMICs. The application of effective, contextually adaptive exit strategies is critical to ensuring the continuity of health gains after external support ends. It emphasises the need for collaborative research focused on long-term impacts and offers concrete recommendations for policy and practice.

**Systematic Review Registration:**

PROSPERO CRD42021236969.

**Supplementary Information:**

The online version contains supplementary material available at 10.1186/s44263-025-00182-6.

## Background

Sustaining the benefits of health interventions following the withdrawal of donor funding is a persistent challenge across global health programmes. Official Development Assistance (ODA) and private philanthropy have historically played critical roles in improving health outcomes in low- and middle-income countries (LMICs), but questions of sustainability, transition to community ownership and maintenance of impact post-funding remain inadequately addressed [1–3]. These concerns have become increasingly urgent in the context of shrinking ODA budgets [4–6], highlighting the need from the start of the initiative for better planning around programme exit, transition, and long-term sustainability.

While research and guidance on responsible exit strategies has grown in the humanitarian sector [7], similar frameworks are far less developed in the international cooperation global health field [8, 9]. Many health interventions are designed without clear transition plans, risking the erosion of gains once external support concludes [10, 11]. Key challenges include misalignment of funder priorities with national health systems, insufficient engagement of local and community stakeholders and limited capacity building efforts [12].

Exit strategies refer to explicit, structured and transparent processes, plans or activities designed to manage the withdrawal of external resources from beneficiary communities [13, 14]. Exit strategies can also manage the transition of programme support or leadership to local actors or systems, with the goal of sustaining the benefits achieved to date [15]. Exit strategies are not singular events fixed to the end of a project; rather, they can be discussed on a rolling basis at different stages of a programme’s lifecycle: before, during or at the point of withdrawal.

Forward-looking exit strategies are often built into the early design and implementation phases of an intervention, anticipating eventual transition or closure from the outset. In other cases, adaptations to exit plans may occur during project implementation as programmes evolve, or more reactively towards the end of the external support when formal closure is imminent [16, 17]. If, however, exit strategies focus exclusively on demonstrating impact at exit, it may jeopardise investment in longer-term sustainability [13]. Across all these scenarios, the overarching aim remains the same: to minimise disruption, maintain gains and empower community ownership following the exit of a foreign donor.

Exit strategies may involve setting clear milestones, as part of a sustainability plan, strengthening institutional capacities and coalition-building with contextual relevance in mind [18–20]. They typically incorporate activities such as knowledge transfer, stakeholder alignment and resource handover to ensure that services do not collapse after donor exit, but continue to operate effectively within the local structures.

Despite the scarcity of literature on exit strategies in global health, there are signs of growing government interest in these. In 2020, for example, the UK Department for International Development (now the Foreign, Commonwealth & Development Office, FCDO) commissioned a helpdesk report on lessons learned on responsible exits from humanitarian interventions [21]. However, research is also needed into exit strategies for health interventions in development cooperation.

This systematic review aims to understand and categorise the common components of exit strategies employed in health interventions funded by ODA and private philanthropy in LMICs. The objective of the review is to compare and contrast existing exit plans, tools and strategies successfully used to facilitate longer-term impact in these contexts.

## Methods

The review is reported according to the Preferred Reporting Items for Systematic Reviews and Meta-Analyses (PRISMA) guidelines [22] (see Additional file 1 PRISMA 2020 checklist) and registered with PROSPERO (ref. CRD42021236969).

### Search strategy

The search strategy was designed to capture a broad range of health intervention studies relevant to exit strategies, including but not limited to mental health contexts. We used inclusive terms related to health programme sustainability, transition and scale-down processes to ensure that studies describing relevant exit or sustainability actions, whether early-stage, mid-course or at closure, would be identified. Database searches were conducted in Embase, Scopus, PubMed, PsycINFO, HMIC Health Management Information Consortium, MEDLINE, Social Policy and Practice, Web of Science and Global Health. A combination of subject headings and keywords across four categories (‘exit strategy’ AND ‘health’ AND ‘intervention’ AND ‘LMIC’) and their synonyms were explored (see Additional file 2 Search Strategy). An initial search was conducted in May 2021, and re-run in January 2023. Results were limited to English, French, Spanish and Portuguese, and articles published since 2000. Forward and backward citation searching was performed on included articles to identify further relevant studies.

### Eligibility criteria

Table 1 describes the eligibility criteria applied to setting, condition, phenomena of interest and publication type domains. The definition of LMIC was drawn from the Organisation for Economic Co-operation and Development (OECD), while the definition of health intervention was based on the World Health Organisation (WHO) International Classification of Diseases 11th Revision [23]. Phenomena of interest are focused on understanding and categorising exit strategies.

**Table 1:** Inclusion and exclusion criteria

Domain	Inclusion criteria	Exclusion criteria
Setting	LMICs*—countries eligible for Official Development Assistance (ODA) as categorised by the Development Assistance Committee of the Organisation for Economic Co-operation and Development (DAC-OECD); countries that are no longer low- and middle-income but were when the intervention was conducted	High-income countries or those not qualifying for ODA were excluded; studies which did not describe interventions in LMICs
Condition	Health interventions**—funded fully or partially by ODA and as defined by the World Health Organisation (WHO), ‘a health intervention is an act performed for, with or on behalf of a person or population whose purpose is to assess, improve, maintain, promote or modify health functioning or health conditions’	Non-health-related interventions (articles on COVID-19 were excluded due to the extraneous and specific nature of the pandemic)
Phenomena of interest	Exit strategies—studies and articles describing an implemented exit strategy, implemented components of an exit strategy, or implemented elements of a sustainability or scalability plan intended to support the transition or continuation of a health intervention’s benefits beyond external funding. Eligible studies could describe activities and processes initiated before, during, or at the point of exit. Included studies should make reference to at least two of the components of exit strategies as laid out in the *Sustainability and Exit Strategies Conceptual Framewor*k by Rogers*** (sustained motivation, resources, capacity and linkages.)	Studies not having implemented a sustainability or exit plan to support the long-term impact of the health initiative after end of programme activities; projects or health initiatives which had failed to design an exit strategy but only referenced estimates or discussed the need for further research or hypothetical case scenarios of next steps if more investment was secured
Publication type	All peer reviewed qualitative and quantitative articles (e.g. case studies, projects or programmes of all designs, data articles)	Grey literature, policy papers, working papers, literature reviews, opinion pieces and narrative only overviews, commentaries; citations only were excluded, abstracts only sources; theoretical and conceptual studies

*LMICs = low- and middle-income countries

**WHO, International Classification of Diseases, Eleventh Revision (ICD-11). 2019/2020

***Rogers, B. L. and Coates, J. (2016). Sustaining Development: A Synthesis of Results from a Four-Country Study of Sustainability and Exit Strategies among Development Food Assistance Projects—Executive Summary. Washington, DC: FHI 360/Food and Nutrition Technical Assistance III Project (FANTA)

During the search strategy development, exit strategies were considered in line with the Rogers and Coates (2016) ‘Sustainability and Exit Strategies Conceptual Framework for Food Programmes’ [13]. Studies were included if they referenced processes reflecting minimum two of the four exit strategy components: (1) sustained motivation; (2) sustained resources; (3) sustained capacity; (4) and sustained linkages.

### Study selection

All search results were imported into EndNote 20.6 [24, 25], deduplicated and imported into Rayyan for screening [26]. Initially, two authors (M.M. and S.T.) independently screened 10% of the titles, abstracts and keywords to identify potentially relevant records for full-text screening. Once 92% threshold for accuracy between screeners was achieved, M.M. screened the remaining titles and abstracts independently. Full-text screening was conducted by M.M. Questions on inclusion eligibility were resolved through discussion between M.M., P.C.G. and T.T.S.

### Data extraction

Data were extracted on descriptive study characteristics (author, year published and country), funder, duration, name and type of intervention and the exit strategy components as described using the Rogers and Coates framework. Additional components which did not fit the framework but were relevant to the phenomena of interest were also considered for inclusion. As this review focuses on the exit-strategy related processes reported in the included studies rather than study outcomes, no outcome data were extracted.

### Quality assessment

The Mixed Methods Appraisal Tool (MMAT) [27] was used to assess the methodological quality of included studies. The tool consists of study-design specific quality assessment questions. Studies using either a qualitative or quantitative design were assessed with five items specific to that study design, whereas mixed methods studies were assessed using questions relating to both the qualitative and quantitative aspects of the study alongside further items pertaining to mixed methods features specifically (i.e. rated using 15 items in total; five items each for qualitative, quantitative, and mixed methods study elements). Two reviewers (M.M. and S.T.) independently assessed the studies’ quality and disagreements were reconciled via consensus (M.M., T.T.S and P.C.G). Each item is rated as ‘yes’ (1 point), ‘no’ (0 points) or ‘unclear’ (0 points). A percentage score was generated for each study, by summating the scores across the relevant quality appraisal items and dividing this by the total number of items assessed.

### Data synthesis

Data on study characteristics were descriptively synthesised. Due to the heterogeneity of the included studies, extracted data regarding exit strategy processes and components were analysed using thematic synthesis [28], using the Rogers and Coates framework of sustaining development as a starting point. First, the extracted details were repeatedly reviewed, and extensive notes were taken. Key processes and components relevant to exit strategies were grouped together and categorised according to theme. Participants’ quotes and authors’ accounts of what steps were taken to achieve longer-term impact, and the design and use of a sustainability or scalability plan, were captured. Data extraction and synthesis were carried out using Microsoft Word and Microsoft Excel following established procedures [29].

## Results

### Study characteristics

**Fig. 1:**
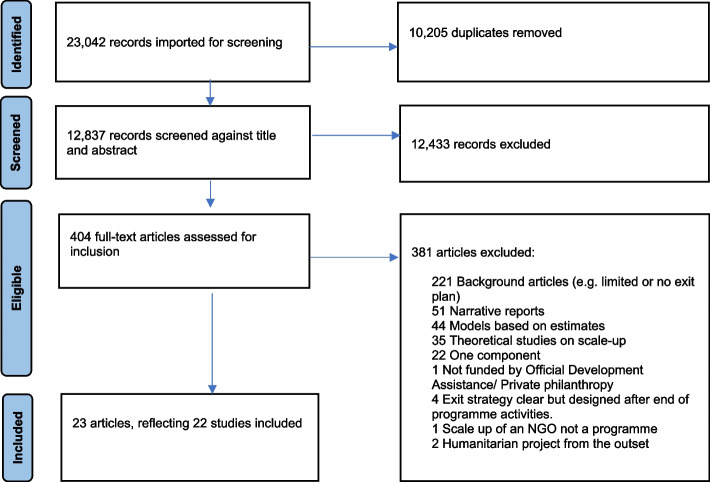
PRISMA flowchart of the included articles

The search resulted in 23,042 records for screening (see Fig. 1). After removing duplicates, 12,837 records were screened by title and abstract, with 404 records eligible for full-text screening. Twenty-three articles were identified for inclusion. All presented a clear example of exiting plans with distinct components which were deemed helpful when building towards impact-oriented project promoted practices.

The 23 included articles correspond to 22 studies (two articles report on the *All Babies Count* (*ABC*) programme [30, 31]). Of the included articles, 47% (*n* = 11) were mixed methods, four percent (*n* = 1) were quantitative and 47% (*n* = 11) were qualitative. Most frequently used qualitative methods were in-depth interviews, focus group discussions, and key informant interviews. Those with a quantitative component (including the mixed methods studies), consisted of routinely collected health data, questionnaires, surveys and evaluation of a pre- and post-design. Characteristics of included studies are reported in Table 2.

**Table 2:** Characteristics of included articles and their exit strategy components

Reference	Country	Funder	Duration	Name and type of intervention	Methods	Exit strategy component
Abas et al. (2016) [33]	Zimbabwe	Donations, Zimbabwe Health Training Support, Medical Research Council, Grand Challenges Canada	2006—ongoing (several stages and versions in between)	Collaborative care mental health intervention; basic cognitive behavioural therapy with an emphasis on problem solving therapy	Mixed-methods	Resource stability; leadership promotion and multi-level partnerships; staff care and capacity; flexibility of exit; monitoring and evaluation
Ario et al. (2019) [34]	Uganda	President’s Emergency Plan for AIDS Relief (PEPFAR), President’s Malaria Initiative, Global Health Security Agenda	Two years for each fellow (programme has been active 2015–2018 since study, and ongoing)	To develop a competent workforce to manage epidemics and improve disease surveillance, especially communicable but also non-communicable diseases	Qualitative	Local champions for post-exit services; staff care and capacity; leadership promotion and multi-level partnerships; shared principles and values; flexibility of exit; resource stability; operational linkages; monitoring and evaluation
Bandali et al. (2021) [35]	17 countries in Africa and Asia	UK Foreign, Commonwealth and Development Office (FCDO)	2018–ongoing	Provides comprehensive sexual and reproductive health services, aiming to improve universal access to quality family planning and safe abortion services (where local laws allow this)	Qualitative	Shared principles and values; staff care and capacity; flexibility of exit; monitoring and evaluation; operational linkages
Bandoh et al. (2019) [36]	Ghana	US Centre for Disease Control funded the first 5 years; since then funded by donors, stakeholders and residents	2007–2017 (ongoing)	Building and strengthening national and local capacity to effectively respond to public health emergencies and mitigating their impact	Qualitative	Flexibility of exit; staff care and capacity; resources stability; monitoring and evaluation
Batti (2014) [52]	Kenya	NGOs, MoH, individuals, Kijabe hospital, churches, World Vision Kenya	N/A	Health centres proving primary health care services	Mixed-methods	Resource stability; monitoring and evaluation; operational linkages
Bennett et al. (2015) [37]	India	The Bill and Melinda Gates Foundation (BMGF)	2003, final transition 2013–2014 until incorporated in Government	Peer education, condom distribution, testing and treatment of sexually transmitted infections, clinical service referral, community mobilization, as well as structural interventions aimed at reducing stigma, violence, and barriers to accessing entitlements	Mixed methods	Staff care and capacity; flexibility of exit; operational linkages; monitoring and evaluation; leadership promotion and multi-level partnerships; shared principles and values
Blanchet et al. (2014) [38]	Ghana	Swiss Red Cross; eye care staff were all employed and paid by their hospital; user fees were only paid for facility-based activities (consultations and cataract surgeries); all outreach activities were free of charge	1996–2006	Intervention to increase uptake of eye care services	Mixed-methods	Flexibility of exit; staff care and capacity; resource stability; monitoring and evaluation
Chowdhary et al. (2022) [39]	Ethiopia	The Nike Foundation first, then Johnson & Johnson	2010–2013 (ongoing)	﻿To provide reproductive health and financial savings curricula to married girls via reflective dialogues in peer-based solidarity groups	Qualitative	Flexibility of exit; leadership promotion and multi-level partnerships; monitoring and evaluation; staff care and capacity
Colom et al. (2018) [40]	Guatemala	Grants and targeted fundraising to key donors. Once cost projections were better refined, costs were largely covered by a percentage of the interest rate that is charged to new clients	2015–2018 (ongoing)	To provide primary care services to female clients of a microfinance institution which provides education to mostly Maya indigenous women	Qualitative	Resource stability; leadership promotion and multi-level partnerships; staff care and capacity; shared principles and values; operational linkages; monitoring and evaluation; flexibility of exit
Fenwick et al. (2009) [32]	Burkina Faso, Mali, Niger, Uganda, Tanzania and Zambia	Bill and Melinda Gates Foundation	2002–2008 (key services sustained or incorporated within health system)	To encourage development of sustainable schistosomiasis and soil-transmitted helminth control programmes in sub-Saharan Africa	Mixed-methods	Flexibility of exit; operational linkages; monitoring and evaluation; staff care and capacity; resource stability
Ghag et al. (2021) [41]	Philippines	Ferring Pharmaceuticals’ Corporate Social Responsibility Programme	2015–2017 implemented (now sustained and ongoing)	To design and implement training programme on multi-professional obstetric emergencies Practical Obstetric Multi-Professional Training (PROMPT)	Qualitative	Local champions for post-exit; resource stability; flexibility of exit; operational linkages; monitoring and evaluation; staff care and capacity; leadership promotion and multi-level partnerships
Ghiron et al. (2014) [42]	Uganda and Kenya	United States Agency for International Development (USAID)	2011–2014 (sustained until 2019) institutionalised in surrounding communities	To reduce threats to population, health and environment while simultaneously increasing access to family planning and maternal, child, sexual and reproductive health services from a rights-based perspective	Mixed-methods	Resource stability; shared principles and values; staff care and capacity; flexibility of exit; leadership promotion and multi-level partnerships; monitoring and evaluation
Hainsworth et al. (2014) [43]	Ethiopia, Ghana, Mozambique, Tanzania and Vietnam	United Nations Population Fund and Pathfinder International	1999–2012 (across 5 projects)	Phased and gradual expansion to scale-up adolescent contraceptive services and incorporate lessons learnt	Qualitative	Flexibility of exit; resource stability; staff care and capacity; leadership promotion and multi-level partnerships; shared principles and values; operational linkages; local champions for post-exit; monitoring and evaluation
MacDonald et al. (2014) [44]	Uganda, Kenya and Tanzania	International Development Research Centre of Canada; Dalhousie Medical Research Foundation; Canadian Child Health Clinician Scientists Program, and private donations (plus in-kind donations; IWK Health Centre, Canadian Paediatric Society; Mbarara University of Science and Technology, Healthy Child Uganda; Makerere University; University of Nairobi; Tanzanian Training Centre for International Health, and Aga Khan University)	2008–2013-ongoing	Aimed at enhancing the research capacity of local frontline healthcare professionals to find local solutions for community health problems that can then influence health programs and/or government policy	Quantitative	Leadership promotion and multi-level partnerships; principles and values of all stakeholders; staff care and capacity; flexibility of exit; local champions for post-exit; monitoring and evaluation; resource stability
Mansour et al. (2010) [45]	Egypt	USAID funding for 1 year; after that sustained by covering costs of materials and transportation by facilitators and participants	2002–2003 with foreign funding (2005–2007 with local/self-funding)	Middle management training to reduce maternal mortality ratio by locally led and sustained leadership development programme	Qualitative	Local champions for post-exit; resource stability; operational linkages; staff care and capacity; flexibility of exit; shared principles and values; monitoring and evaluation; leadership promotion and multi-level partnerships
Nahimana et al. (2021) [30]	Rwanda	Ministry of Health and Partners In Health (PIH); Doris Duke Charitable Foundation’s Africa Health Initiative	2005–ongoing	All Babies Count (ABC), a district-wide quality improvement project including mentoring and improvement collaborative designed to improve quality and reduce neonatal mortality	Mixed-methods	Flexibility of exit; leadership promotion and multi-level partnerships; local champions for post-exit; staff care and capacity; monitoring and evaluation
Prasad et al. (2022) [46]	Tanzania	Bloomberg Philanthropies, Foundation H&B Agerup, Blue Lantern, Merck for Mothers, the Swedish International Development Cooperation Agency, and the Swedish Postcode Foundation	2006–2019	Decentralising high-quality maternal and reproductive health services to lower-level facilities in remote, low- resource settings	Mixed-methods	Shared principles and values; flexibility of exit; local champions for post-exit; monitoring and evaluation; leadership promotion and multi-level partnerships; resource stability
Salehi et al. (2021) [47]	Ghana	Global Affairs Canada and the SickKids Foundation with in-kind contributions provided by Ghana Ministry of Health	2016–2020 (ongoing)	National paediatric nurse training program	Mixed-methods	Staff care and capacity; monitoring and evaluation; leadership promotion and multi-level partnerships
Torpey et al. (2010) [50]	Zambia	PEPFAR through USAID	2004–2009	To facilitate operational sustainability in implementing HIV clinical services in Ministry of Health facilities	Qualitative	Operational linkages; staff care and capacity; leadership promotion and multi-level partnerships; flexibility of exit; monitoring and evaluation; resource stability; local champions for post-exit services; shared principles and values
Waiswa et al. (2021) [51]	Uganda	The Einhorn Family Foundation and the Social Initiative, both from Sweden	2013–2016 (post-exit institutionalisation of practices ongoing)	Improvements in maternal and new-born outcomes and scale-up of quality improvement process at hospitals and health centres	Mixed-methods	Operational linkages; shared resource stability; flexibility of exit; staff care and capacity; local champions for post-exit; leadership promotion and multi-level partnerships; monitoring and evaluation
Werdenberg et al. (2018) [31]	Rwanda	Doris Duke Charitable Foundation’s Africa Health Initiative	2005–ongoing	All Babies Count (ABC), a district-wide quality improvement project including mentoring and improvement collaborative designed to improve quality and reduce neonatal mortality	Mixed-methods	Shared principles and values; leadership promotion and multi-level partnerships; flexibility of exit; monitoring and evaluation; staff care and capacity
Wickremasinghe et al. (2021) [48]	Nigeria	Bill & Melinda Gates Foundation	2016–2019, and ongoing within government	New cadre of community health worker, providing maternal, new-born and child health-related messages, basic healthcare and making referrals to health facilities	Qualitative	Flexibility of exit; operational linkages; monitoring and evaluation; staff care and capacity; resource stability; shared principles and values
Zakumumpa et al. (2021) [49]	Uganda	PEPFAR	2013–2018, and ongoing	Develop a competent workforce to manage epidemics and improve disease surveillance, especially communicable but also non-communicable diseases	Qualitative	Local champions for post-exit; resource stability; shared principles and values; flexibility of exit; operational linkages; monitoring and evaluation; leadership promotion and multi-level partnerships

Five of the included studies conducted research in multiples countries. Uganda (*n* = 7), Tanzania (*n* = 5) and Ghana (*n* = 4) were the most studied countries. Most studies (*n* = 18) reported findings from a single country.

All articles but one (34) were published in the last 10 years, with 47% (*n* = 11) published in the last 5 years (2019–2023). More than 80% (*n* = 18) of the studies reported strategies for longer term impact (10 + years) and/or evolved versions of ongoing original programme activities [30, 32–49]. Most studies (59%) focused on maternal and new-born care interventions (*n* = 7) and sexual health and family planning (*n* = 6). The remaining studies focused on non-communicable diseases (NCDs) and mental health conditions, public health emergencies, primary health services, eye care services, research capacity for healthcare professionals and HIV and antiretroviral therapy.

All studies implemented plans and tools for longer-term impact of their projects but only four articles (18%) specifically mention an *exit strategy* verbatim [32]. Five articles (22%) discussed applying a pre-existing sustainability or innovation framework to their projects from the start (such as the Challenge Model and the Leading and Managing Practices Framework [45] and the Consolidated Framework for Implementation Research (CFIR) [42, 43]). The most common funders were United Stated Agency for International Development (USAID), the Bill and Melinda Gates Foundation, and the President’s Emergency Plan for AIDS Relief (PEPFAR).

In terms of methodological quality, qualitative studies met on average 4.5 out of five criteria. The single quantitative study met all five quality criteria, and mixed method studies met on average 12 of 15 criteria (see Table 3). The most common limitation within the qualitative studies was the uncertainty that findings might not be adequately derived from the data. The mixed methods studies were mainly limited by the lack of access to sufficient quantitative data in the setting. This pattern was noticed when it was frequently unclear if the sample was fully representative of the target population. At times, it was also unclear if the different components of the mixed methods studies adhered to the quality criteria of each tradition of the methods involved.

**Table 3:**
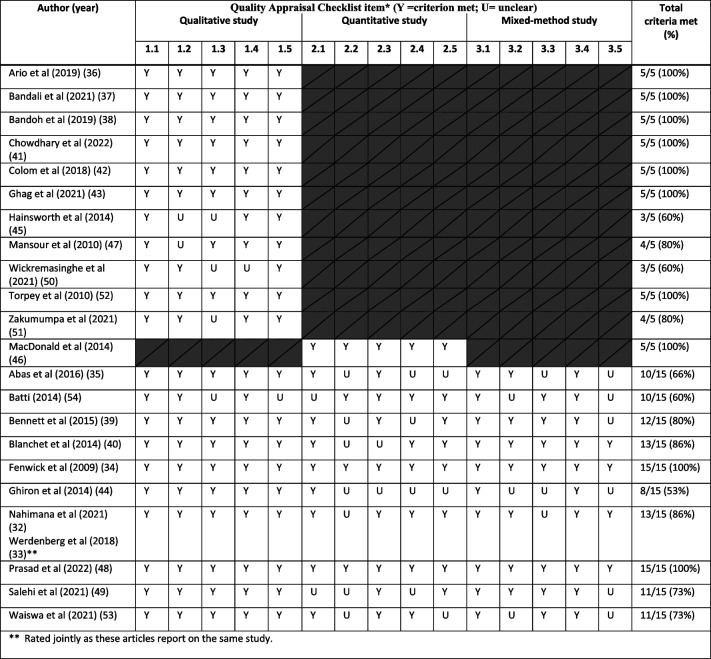
Scores on the Mixed Methods Appraisal Tool (MMAT) of all 23 articles, representing 22 studies

### Categorising common components of exit strategies

**Fig. 2:**
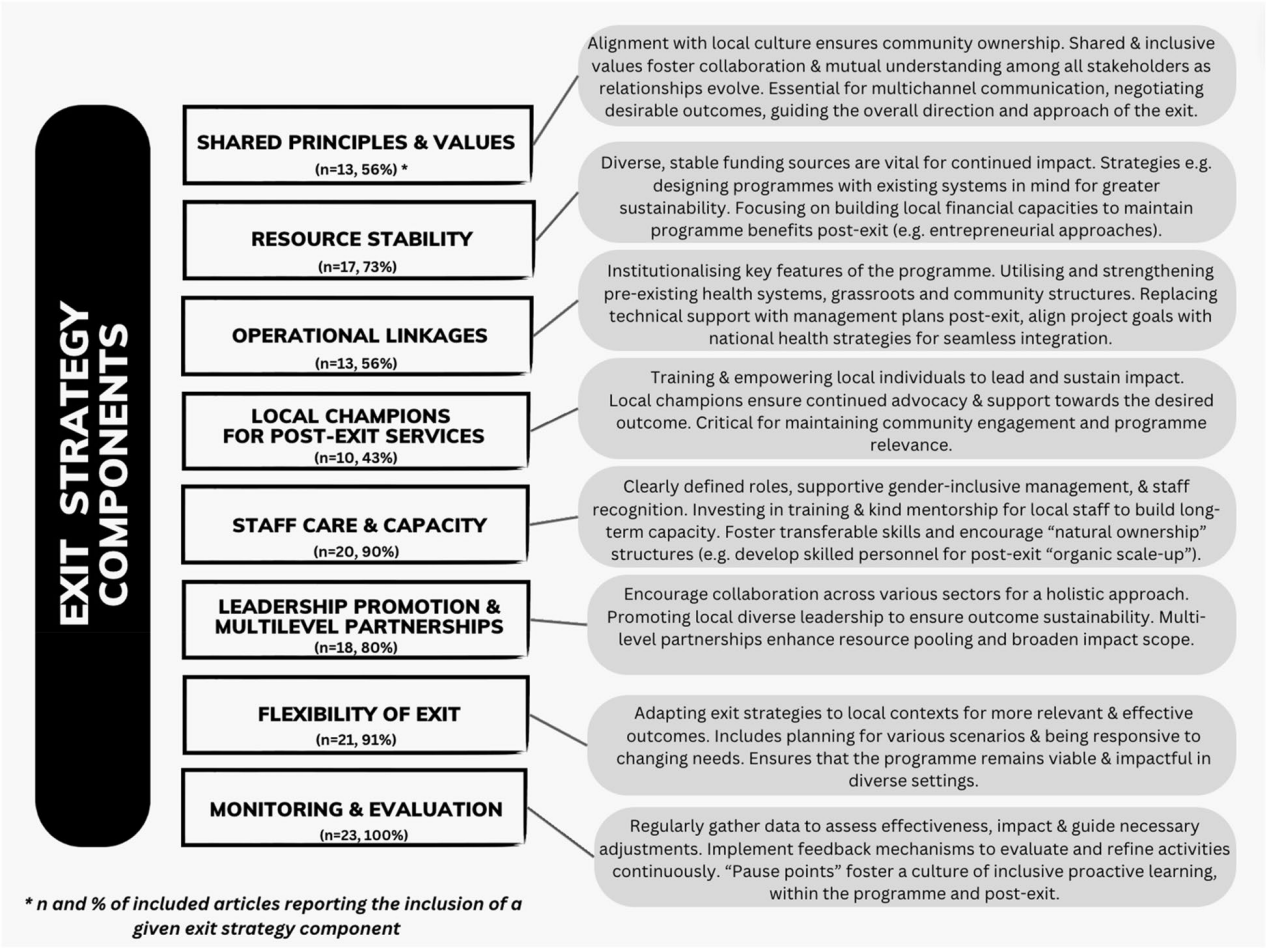
Exit strategy components described in the included literature

The included studies illustrated a variety of ways to increase the likelihood of responsibly exiting a programme and sustaining its impact. While the elements and processes differed between studies, eight components were commonly included: (i) shared principles and values; (ii) resource stability; (iii) operational linkages; (iv) local champions for post-exit services; (v) staff care and capacity; (vi) leadership promotion and multi-level partnerships; (vii) flexibility of exit; and (viii) monitoring and evaluation (see Fig. 2).

### Shared principles and values

A country-driven approach was critical for the scheme’s sustainability: stakeholders went to great lengths to avoid the scheme being introduced in a top-down, donor prescribed fashion, which is a common problem with donor-funded interventions that limits their sustainability [48].

More than half of the articles (56%; n = 13) emphasised the importance of an initial stakeholder mapping and buy-in. Identifying relationships between different actors can help understand their interests, values and how those evolve before, during and after the intervention. Specifically, helpful structures include frequent use of functional communication channels [50], exit leadership and collaboration levels [31, 40, 49], information sharing to assess local needs, and creating trust [37, 46]. A mismatch between outcomes prioritised by communities (e.g. social activities, healthcare services for at risk populations) versus governments or funders (e.g. condition focussed services) was identified as a barrier to sustainability, leading to misunderstandings and reduced impact [37, 42]. Negotiating desirable transition outcomes can harmonise objectives across stakeholders, and an enhanced clarity on transition aims also supported evaluation efforts [26–28, 30, 36, 38, 39, 44]. For example, in a study assessing the development of a competent workforce to manage epidemics and disease surveillance in Uganda and India [34], differences in implementation impacted sustainability. In the non-degree awarding scheme in Uganda fellows already held a postgraduate degree. They spent more time working on programme-oriented projects addressing national health needs, and less time in didactic courses. This reinforced the Ugandan government’s goal of retaining the fellows within the national health system post-exit. In India, however, where the same initiative was structured as a postgraduate degree awarding programme, the short-term outcome of the participants was to earn good grades, graduate from the prestigious degree and study aboard.

### Resource stability

Scaling up depends on having some key resources, but it also depends to a large degree on how those resources are managed [45].

Most articles (73%, *n* = 17) discussed the importance of having a pool of stable resources to ensure sustained impact. Pilot studies often fall short in generating sustainable impacts; this is attributed to the specialised resources needed during the testing phase, which are hard to replicate at scale [42]. Leveraging readily available resources has been recommended [51]. Free provision of resources can be a double-edged sword; while it aids in initial setup, it can jeopardize impact and community ownership post-exit when suitable substitute incentives are neglected [38, 41, 45, 48]. To address this, diversifying funding sources has been encouraged as a more organic way for a programme to exit. Strategies include ‘piggybacking’, where an intervention is incorporated into existing systems like healthcare or social welfare, and ‘graduating’, where certain milestones trigger the withdrawal of external resources but continue with critical support measures (e.g. transforming a healthcare centre into a mobile clinic) [36, 40, 46, 51, 52]. In Mozambique, after laying a solid foundation for both expansion and institutionalisation, a programme on adolescent contraceptive services effectively diversified its exit to meet community-requested adolescent health needs [43]. Another instance in East Africa saw MicroResearch substituting small loans with non-repayable research grants to healthcare professionals keen on tackling community health issues through their own research [44].

### Operational linkages

‘proof of concept studies’ […] require special resources and support to ensure proper implementation during testing, which subsequently cannot be replicated on a larger scale. Awareness is growing that in order to learn whether and how interventions can be successfully scaled up, they must be tested with the people and in the systems that will be responsible for them, thereby providing ‘proof of implementation’ [42].

Operational linkages accounted for 56% (*n* = 13) of the articles. A notable discrepancy on implementing partner and funder involvement for operational set-up was highlighted in five studies, encompassing aspects such as building financial management capacities and programmatic design [43, 46, 48, 50, 52]. To counter this, some interventions sought localised operational models. For instance, a programme focussed on building a resilient and sustainable health system in Uganda, was run entirely by the local implementing team. The external funder contributed only through a technical advisor from the US Centre for Disease Control who was not part of the main team [34]. Nonetheless, there remains a tendency to set up parallel structures in cases where the existing system is not functioning well, rather than undertaking system strengthening as an integral part of the exit strategy [37, 42, 50]. In Guatemala, a microfinance institution aspiring to offer healthcare services chose to partner with the Wuqu'Kawoq (Maya Health Alliance), instead of developing a separate internal healthcare programme. As an existing health provider, Wuqu’ Kawoq was widely used by local indigenous women, so setting up supervisory and technical structures with long lasting impact was feasible [40]. Operational linkages can sometimes be the missing sustainability element. In Ghana, after successfully training paediatric nurse specialists to deliver high-quality care, some graduates saw their skills deteriorating after 14 months [47]. Although partnerships with local health authorities and the Ghana College of Nurses were established, the operational linkages with the hospital administration and leadership led to underutilisation of specialised skills.

### Local champions for post-exit services

At an institutional level, we found that the presence of transition ‘champions’ at multiple levels including within district governance systems but also at the facility level was a key enabler of increasing budgetary allocations for expanding the health workforce in Uganda [49].

Forty-three percent (*n* = 10) of the studies highlighted local champions for post-exit service delivery. Community acceptance breeds motivation [34, 36, 37, 41–43, 46, 48, 49, 51]. In the development and delivery of a multi-professional obstetric emergency training programme in the Philippines, local advocates made addressing organisational challenges more manageable [41]. The role of local health workers and leaders in the design, sustainability and scalability of interventions is key. In Uganda, harnessing available resources to develop local advocates who then lead, integrate, build on and sustain simple, low-cost health packages has been effective. This is done through collaborations between health managers and project staff [51]. Where support is lacking, introducing new concepts (such as quality assurance and quality improvement (QA/QI)) can lead to resistance and suspicion of ‘policing’ among healthcare workers at facility level [50]. To remedy this, ongoing mentorship and supportive supervision was crucial in change management required to build capacity and maintain understanding for QA/QI process.

### Staff care and capacity

I organized a ward meeting with my staff. I have taught them emergency management, pain, NG [nasogastric] tube. … I gave them the rationale and now they can practice in my absence [47].

Staff care and capacity are critical components emphasised by nearly 90% of the studies (*n* = 20). When scaling up education for paediatric nurse specialists in Ghana, staff care formed the cornerstone of the exit strategy [47]. Utilising a phased, capacity-building model that pairs local mentors with national-level counterparts from the outset is instrumental for success [51]. Stakeholder motivation and good will can support low-cost or no-cost interventions in the short-term. In Zimbabwe, health promotion is viewed as a ‘caring’ profession so recruitment for a community based psychological interventions was initially conducted on a voluntary basis [33]. However, in the long run, this led to staff burnout and high levels of turnover. Vital factors for staff care include ‘natural ownership’ and ‘organic scale-up’ (e.g. ability to influence ongoing work), understanding of one’s role, supportive and flexible management style and tangible recognition of work (e.g. pay, promotion, benefits) [35, 37, 39, 41, 45, 47, 50]. A smooth transition becomes challenging when there is ambiguity regarding the continuation of services post foreign-support withdrawal. This lack of clarity can foster internal staff conflicts, potentially jeopardizing programme sustainability [38].

### Leadership promotion and multi-level partnerships

Gender equity was seen in the leadership of the most successful MicroResearch teams in East Africa [44].

Eighty percent (*n* = 18) of the publications underscore the importance of leadership promotion and multi-level partnerships. They recognise that health programming does not operate in a vacuum, and that siloed approaches to development are limited in the impact they can have [44, 46]. A project on improving maternal and new-born care in Tanzania exemplified this by being organically developed to fit regional health needs [46]. Heavily supported and leveraged by government via decentralised partnerships with multiple organisations, the initiative grew over decades. Regular evaluations were employed, expanding the definition of leadership to include bottom-up perspectives, and utilising flexible funding as key success factors. In contrast, a neonatal mortality reduction project in Rwanda highlighted the potential pitfalls of ambitious but inexperienced leadership [30]. Leaders were committed but often lacked the managerial and technical skills, which led to a diffusion of focus and affected the project’s momentum post-implementation. In Uganda, balanced partnerships between local and foreign governments and academia were instrumental in establishing the Uganda National Institute of Public Health as a unique directorate for training, employing fellows dedicated to health system strengthening [34]. But when government involvement is lacking, it is crucial to develop a transparent exit strategy early on. Such strategies usually involve transferring project support from the implementing organisation to the Ministry of Health (MoH) [50]. This was exemplified in a study that successfully transitioned health workers’ contracts to government payrolls. Targeted donor aid helped overcome systemic barriers in decentralised settings where the government had limited capacity [49]. To prepare for this transition, conducting district wage bill analyses and engaging multiple sectors, including the Ministry of Finance, were essential for the incoming implementation responsibilities [34, 40, 44].

### Flexibility of exit

This tool is easier to use because it gives you a head start. It already shows all the possible ways, clear and simple. For me, personally, the projects before were not flexible, it was rigid, it had to be done, I couldn’t change it …With WISH, we had the opportunity to propose changes. This is very important, it is more suitable to the context. It’s not ‘top-down’ but it really fits into the context of the country [35].

Programmes with successful longer-term positive impacts have shown the ability to flexibly adapt to context; in our review, 91% (*n* = 21) of the articles confirmed that. Uncertain political, environmental, societal and economic fluctuations can hinder this adaptability, especially if rigid adherence to funder-imposed timelines and goals are prioritised [30, 32–35, 37–39, 41, 43, 45, 46, 48, 49]. An FCDO programme on sexual/reproductive health implemented across 28 countries, was designed using an outcome-based approach, as opposed to identifying a specific intervention with set activities [35]. The programme pre-approved nine different ‘pathways’, each comprising a sustainability goal, annual indicators, steps and milestones. This approach enabled country teams to select the most context-appropriate route to their desired outcomes. Other programmes have made flexibility their priority by skilfully using marketing and exit strategies to adapt to context momentum [33]; or maintaining a flexible list of activities while adhering to stable programme objectives to yield long-lasting benefits [38]. One study reported mishandling a political transition so project activities could not be absorbed by the new government [48], and another highlighted funders'flexibility in reallocating resources for unanticipated activities [46].

### Monitoring and evaluation

Our interest in transition M&E [monitoring and evaluation] is based upon the belief that, if well designed and implemented, transition monitoring can provide early warning to stakeholders of problems in the transition process that need to be addressed and, over the longer term, evaluation can reassure both development partners and governments that the effects of initial investments have been sustained [37].

The unanimous consent among the articles included (*n* = 23) is that robust monitoring and evaluation (M&E) practices are critical for successful impact and long-term sustainability of health outcomes (see Table 2). Conventional M&E frameworks can miss the mark, focussing on programme progression rather than the appropriateness of the path [30, 35, 53]. However, well-designed monitoring offers an early warning system for potential issues, so challenges can be addressed proactively. Jointly agreed-upon quarterly and annual M&E measures have been instrumental in extracting practical lessons and aligning stakeholders on the way forward [34, 46, 49, 51]. For instance, a large-scale HIV/AIDS prevention initiative in India utilised an array of quantitative metrics (comparable to institutionalisation indicators), assessing pre- and post-transition readiness, the continuity of essential practices and services coverage, to gauge their programme’s resilience [37]. In Tanzania, a project focused on improving maternal and reproductive health had the ability to respond to real-time M&E data, which proved invaluable [30, 46]. Demand-generation activities, not part of the original plan, were incorporated as a direct result of interim evaluation insights. This type of adaptability stresses the importance of frequent monitoring, supported by either external or internal evaluations, and the use of real-time automated reporting systems [30–32, 35, 37, 40, 43, 48]. The inclusion of ‘pause points’ throughout the programme cycle and especially towards the exit allowed for timely reflections and necessary adjustments, aligning the intervention’s evolution with the shifting on-the-ground-realities. These ‘pause points’ foster a culture of proactive learning, encouraging stakeholders to engage in reflection loops that inform implementation and facilitate the adaptation of strategies [35, 48]. Such an approach not only ensures that programmes remain relevant and responsive but also empowers local and community stakeholders to take ownership of the intervention’s future.

## Discussion

Often accelerated by investment from ODA and private philanthropy, the expansion of global health programming has led to a proliferation of time-limited, donor-initiated interventions that frequently lack clear plans to strengthen long-term in-country capacity for implementation [54, 55]. Without direction and cohesive coordination, suspension of services, erosion of public health gains and loss of sustained impact become recurrent [56–61]. This systematic review provides significant insight into the need for structured exit strategies for health initiatives funded by ODA and private philanthropy in LMICs. Eight emerging components of exit strategies for the successful transition of health intervention implementation were identified: shared principles and values, resource stability, operational linkages, local champions for post-exit services, staff care and capacity, leadership promotion and multi-level partnerships, monitoring and evaluation and context-sensitive flexibility of exit. These components are not always distinctly separate; overlaps, such as between local champions and staff care, indicate their interconnected and interdependent nature.

Each one of the components highlights the importance of collaborative approaches that involve local and community stakeholders in the design, implementation and evaluation of health interventions. To maximise the chances of success after a funded programme concludes, it is crucial to engage local partners who possess the necessary leadership and management skills to advocate for sustained impact. Achieving this requires aligning the intervention and its delivery strategies with the local and community stakeholders’ values, priorities, goals and knowledge. Placing local buy-in at the core of research methods is an approach used by participatory action research [62], community health worker programmes [11], integrated community case management [63] or co-creation and co-design [64]. A method worth mentioning is human-centred design (HCD) [65]. HCD is a creative approach to problem-solving that starts with understanding the needs, behaviours and experiences of the people for whom a solution is being designed. It involves users throughout the design process to ensure the outcomes meet their needs and are usable in their context. To better support researchers and funders, careful consideration and application of an exit strategies framework designed alongside local and community stakeholders could help maximise the chances of sustained impact after an externally funded intervention comes to an end.

Existing literature on donor support also indicates that many funded initiatives are not supported by governments and health systems due to a lack of local resources, making them “boutique” projects with limited effectiveness [48, 66, 67]. To a large extent, these issues show that the impacts of donor transitions on health systems are both understudied, and as our findings suggest, also inadequately assessed. Funders and researchers should avoid creating interventions that, despite being successful in pilot or feasibility stages, cannot be sustained by local communities due to resource or capacity constraints. A recent scoping review on health systems revealed that most funders-authored publications (such as reports and blogs) bypass formal peer review processes [61]. These publications frequently address leadership, governance and health financing, often overlooking the impact on health outcomes. While responsibility for managing risk factors applies to all stakeholders involved, the critique typically targets the financing shortcomings of national leadership. To illustrate, the substantial funding for HIV/AIDS projects in Uganda of early 2000 s by PEPFAR ($94 million) and Global Fund to Fight AIDS ($150.6 million) went around the Ugandan national health strategy by creating parallel management systems which undermined the government’s ownership and equity long-term [68]. Focussed on providing the much needed immediate relief, funders and researchers failed to incorporate a plan for long-term institutional development, a strategy which would have been better aligned with the principles of harmonised development cooperation of the 2005 “Paris Declaration on Aid Effectiveness” [69, 70]. If an exit strategy had been negotiated early on during the design and implementation phases of the initiative, short-term and long-term outcomes of the intervention could have been considered together, so one is not built on the expense of the other.

Uganda is not the only case where global health actors and local governments are misaligned. Regionally, our review found a preponderance of studies from East Africa (led by Uganda, Tanzania, Ethiopia and Kenya, and followed by Ghana in West Africa). This reflects OECD reporting on health ODA’s focus on sub-Saharan Africa (totalling US$11.9 billion) [71]. Notably, the Bill and Melinda Gates Foundation’s philanthropic efforts in the region have concentrated on health and reproductive health since 2018 [72]. However, according to a recent UN report, a disconnect remains between funding for maternal and new-born care and the actual empowerment of women in their sexual and reproductive health decisions [73]. This gap highlights a universal challenge in exit strategies for health: local governments often view foreign projects as temporary fixes rather than as catalysts for developing sustainable responses. This perspective is indicative of a systemic mismatch between policy development and its execution [74].

Without societal and political intervention to empower the most vulnerable, no amount of sporadic outside support will be sufficient to address the gaps left behind. Due to their visibility and high impact on the health system, policymakers, funders and practitioners should prioritise the development of exit strategies that are intrinsically linked to local systems, ensuring that interventions can be absorbed and sustained by local infrastructures. Programme developers should focus on establishing and strengthening multi-level partnerships and leadership structures from the onset, ensuring that programmes are not only fit for the immediate context but are flexible and adaptable to the evolving financial, contextual and healthcare realities. While the timing of when specific components (e.g. forming partnerships or setting up monitoring systems) are initiated may vary, the process of designing an exit strategy involves addressing all components from the beginning. This approach urges practitioners to consider sustainability holistically, rather than treating entry and exit as separate stages.

The current gaps in research highlight a pressing need for tailored exit strategies which can provide comprehensive insights into both immediate and sustained impacts of the implemented research projects. Future research should aim to fill the current gaps by incorporating robust, outcome-focused monitoring and evaluation frameworks that transcend traditional input and activity metrics, providing a clearer pathway for all stakeholders involved on how health outcomes can be sustained. The literature would benefit from more interdisciplinary publications that integrate economic, social and health perspectives with community and managerial expertise to offer a more holistic understanding of long-term health structures.

A critical takeaway for implementers is the importance of fostering community ownership and building local, in-country capacity, which is often the linchpin of enduring success. For instance, community health committees and boards can influence health outcomes [75]; by intertwining health and economic empowerment, maternal and child health can be improved while increasing women’s access to better livelihoods [76]; and community forums, feedback mechanisms and regular review meetings with stakeholders can be integrated in collaborative frameworks for continuous learning [77]. Programmes must be designed with the end in mind, considering local resources and structures that will remain post-exit. This forward-thinking approach, coupled with flexible funding mechanisms that respond to on-the-ground needs identified through continuous M&E, is vital for creating lasting health improvements [78, 79].

The relatively small number of studies found that describe exit strategies and the differences in terms used highlight that despite an increase in attention, there is either little consideration of what happens in communities after the end of a funded programme and/or researchers and/or journals are uninterested in publishing this information. Further work is needed to develop an exit strategies framework to support global health researchers. We acknowledge that while the search strategy was broad and designed to capture a wide range of health intervention exit and sustainability practices, it is possible that some context-specific strategies, particularly unpublished or non-English sources, may not have been fully captured. We also acknowledge that exit strategies as a concept are only as effective as the environment in which they are developed. For instance, the absence of enabling conditions—such as aligned stakeholder priorities, adequate resources and systemic readiness—can make even the most robust exit strategies insufficient. Another limitation is that some programmes, while well-intentioned, may be inherently unsustainable from the beginning due to factors such as overdependency on foreign resources, lack of needs assessments of local contexts or unrealistic scaling up expectations. This does not negate their value but highlights the need for greater scrutiny during the design phase and implementation to manage expectations, potential post-funding impacts and sustainability potential. Finally, we will emphasise the importance of realistic goal setting. For programmes that are unlikely to achieve full sustainability, we suggest the value of designing for time-limited impact, with clear exit strategies for transitioning lessons learnt to other local actors or systems, rather than viewing unsustainability or lack of scalability as failures.

To our knowledge, this is the first systematic review to thoroughly examine the use of exit strategies and the withdrawal of external resources from health programmes in LMICs, while still aiming to preserve and enhance the positive outcomes achieved without undermining their long-term impacts. More than half of the included articles were published in the last 5 years (*n* = 13, 56%). This trend indicates a growing interest in the area. The strengths of this systematic review reside in its timeliness and potential for impact by filling an important gap in the literature. Within the implementation science field, our findings on exit strategies can shape the thinking of funders, policy makers, research institutions and implementation actors.

## Conclusions

Sustained long-term impact beyond the actively funded period of any project is not a serendipitous outcome but a deliberate product of strategic planning and local engagement. This systematic review has highlighted the critical importance of well-designed exit strategies in the realm of international health programme cooperation. The interdependencies of the common components of published exit strategies (shared principles and values, resource stability, operational linkages, local champions for post-exit services, staff care and capacity, leadership promotion and multi-level partnerships, monitoring and evaluation and context-sensitive flexibility of exit) illustrate the complex nature of sustainability in health and the need for a holistic, systematic approach to programme design and implementation.

Exit strategies should not be an afterthought but a core consideration from the earliest stages of the conception and design of an intervention, throughout its implementation and closure. Embracing this principle is crucial for achieving the long-term impact of sustainability in health in limited resource settings. As needs and funding priorities in global health continue to evolve, the onus is on funders, researchers, and policymakers alike to ensure that the investments made today do not merely vanish tomorrow, but instead seed the ground for resilient health systems in LMICs.

## Supplementary Information

Supplementary Material 1. PRISMA 2020 Checklist.

Supplementary Material 2. Generic Search Strategy – Key word concepts.

## Data Availability

All data supporting the findings of this study are available within the paper and its Supplementary Information.

## Abbreviations

FCDO: Foreign, Commonwealth and Development Organisation
GMH: Global Mental Health
HIC: High-income country
LMIC: Low-and middle-income country
M&E: Monitoring and evaluation
MoH: Ministry of Health
NCD: Non-communicable diseases
NGO: Non-governmental organisation
NIHR: National Institute for Health and Care Research
NTD: Neglected tropical diseases
ODA: Official Development Assistance
OECD: Organisation for Economic Cooperation and Development
QA/QI: Quality assurance/Quality improvement
SDG: Sustainable development goal
UN: United Nations
UNIPH: Uganda National Institute of Public Health
USAID: United States Agency for International Development
US CDC: United States Centre for Disease Control
WHO: World Health Organisation
