# Supplementary material for: Exit strategies for health interventions in low- and middle-income countries: a systematic review
Source: BMC Glob Public Health. 2025 Jul 21;3:63. doi: 10.1186/s44263-025-00182-6 (PMC12281693; doi:10.1186/s44263-025-00182-6)
Supplement: Supplementary file 2 — Supplementary Material 2. Generic Search Strategy – Key word concepts. [file 44263_2025_182_MOESM2_ESM.docx]

**Generic Search Strategy – Key word concepts**

| **Exit Strategy (AND)** | **Health (AND)** | **Intervention (AND)** | **Low- and Middle Income Countries** |
| --- | --- | --- | --- |
| “Exit strateg*” OR “Sustain*” OR  “Scal*” OR  “Scal*-up” OR  “Upscal*” OR  “Uptake” OR  “Phase-out” OR  “Phase-over” OR “Outcome*” OR  “Impact*” OR  “Before and after” OR  “Transition” OR  “End of ” OR  “Long-term” OR  “Knowledge transfer” OR “Evaluation” OR “Improvement” OR “Closure” OR “Dissemination” OR “Diffusion” OR  “Research-to-practice” OR “Capacity building” OR “Capacity-building” OR “Theory of change” OR “Replication” OR “Adaptation” | “Global health” OR  “Public health” OR  “Healthcare” OR  “Mental health” OR  “Health services research” OR “Health economics” OR  “Health policy research”  “Community health” OR  “Primary healthcare” OR  “Health systems” OR  “Health facilities” OR  “Community health workers” OR  “Preventative health” OR  “Reproductive health” OR  “Rural health services” OR  “Health promotion” OR  “Health outcomes” OR  “Maternal health” OR  “Child health” OR  “HIV / AIDS” OR  “Tuberculosis / TB” OR  “Malaria” OR  “Non-communicable diseases” OR  “Depression” OR  “Anxiety” OR  “Disorder*” OR  “Adolescent health” OR  “Disease burden” | “Intervention” OR  “Intervention development” OR “Project” OR “Program*” OR “Initiative” OR “Study design” OR  “Scheme” OR  “Trial” OR  “Action plan”  “Implementation” OR  “Service delivery” OR  “Treatment” OR  “Therapy” OR  “Development program*” | “Developing countr* OR  “Developing nation* OR  “Less developed countr*” OR  “Least developed” OR  Resource-limited setting” OR  “Global South” OR  “Emerging econom*” OR  “poor countr* OR  “under developed countr* OR  “Africa” OR  “Asia” OR  “South America” OR “Latin America” OR  “Central America” OR “LMIC” OR  “Low resource” OR  “Emerging economies” OR  “Middle-income countries” OR  “Low-income countries OR  “Upper-middle income countries  OR  "Burundi" OR "Madagascar" OR "Syrian Arab Republic" OR "Central African Republic" OR "Malawi" OR "Togo" OR "Chad" OR "Mali" OR "Uganda" OR "Congo, Dem. Rep" OR "Mozambique" OR "Yemen, Rep." OR "Eritrea" OR "Niger" OR "Ethiopia" OR "Rwanda" OR "Gambia, The" OR "Sierra Leone" OR "Guinea-Bissau" OR "Somalia" OR "Angola" OR "Honduras" OR "Papua New Guinea" OR "Bangladesh" OR "India" OR "Philippines" OR "Benin" OR "Jordan" OR "Samoa" OR "Bhutan" OR "Kenya" OR "São Tomé and Principe" OR "Bolivia" OR "Kiribati" OR "Senegal" OR "Cabo Verde" OR "Kyrgyz Republic" OR "Solomon Islands" OR "Cambodia" OR "Lao PDR" OR "Sri Lanka" OR "Cameroon" OR "Lebanon" OR "Tajikistan" OR "Comoros" OR "Lesotho" OR "Tanzania" OR "Congo, Rep." OR "Mauritania" OR "Timor-Leste" OR "Côte d'Ivoire" OR "Micronesia, Fed. Sts." OR "Tunisia" OR "Djibouti" OR "Morocco" OR "Uzbekistan" OR "Egypt, Arab Rep." OR "Myanmar" OR "Vanuatu" OR "Eswatini" OR "Nepal" OR "Vietnam" OR "Ghana" OR "Nicaragua" OR "West Bank and Gaza" OR "Guinea" OR "Nigeria" OR "Zambia" OR "Haiti" OR "Pakistan" OR "Zimbabwe" OR "Albania" OR "Equatorial Guinea" OR "Moldova" OR "Algeria" OR "Fiji" OR "Mongolia" OR "Argentina" OR "Gabon" OR "Montenegro" OR "Armenia" OR "Georgia" OR "Namibia" OR "Azerbaijan" OR "Grenada" OR "North Macedonia" OR "Belarus" OR "Guatemala" OR "Paraguay" OR "Belize" OR "Indonesia" OR "Peru" OR "Bosnia and Herzegovina" OR "Iran, Islamic Rep." OR "Serbia" OR "Botswana" OR "Iraq" OR "South Africa" OR "Brazil" OR "Jamaica" OR "St. Lucia" OR "China" OR "Kazakhstan" OR "St. Vincent and the Grenadines" OR "Colombia" OR "Kosovo" OR "Suriname" OR "Costa Rica" OR "Libya" OR "Thailand" OR "Cuba" OR "Malaysia" OR "Tonga" OR "Dominica" OR "Maldives" OR "Türkiye" OR "Dominican Republic" OR "Marshall Islands" OR "Turkmenistan" OR "Ecuador" OR "Mauritius" OR "Tuvalu" OR "El Salvador" OR "Mexico" |
